# Is Antimicrobial Treatment Effective During Therapeutic Plasma Exchange? Investigating the Role of Possible Interactions

**DOI:** 10.3390/pharmaceutics12050395

**Published:** 2020-04-25

**Authors:** Łukasz J. Krzych, Marcelina Czok, Zbigniew Putowski

**Affiliations:** 1 Department of Anesthesiology and Intensive Care, School of Medicine in Katowice, Medical University of Silesia, Katowice, Poland; 14 Medyków Street, 40-752 Katowice, Poland; 2 Students’ Scientific Society, Department of Anesthesiology and Intensive Care, School of Medicine in Katowice, Medical University of Silesia, Katowice, Poland; 14 Medyków Street, 40-752 Katowice, Poland

**Keywords:** therapeutic plasma exchange, plasmapheresis, antibiotics, antimicrobial treatment, drug–drug interactions

## Abstract

Antimicrobial treatment during therapeutic plasma exchange (TPE) remains a complex issue. Recommendations based on a limited number of experimental studies should be implemented in clinical practice with caution. Effective management of infections due to plasma or albumin-related interactions, as well as impaired pharmacokinetics, in critical illness is difficult. Knowing the pharmacokinetics of the drugs concerned and the procedural aspects of plasmapheresis should be helpful in planning personalized treatment. In general, possessing a low distribution volume, a high protein-binding affinity, a low endogenous clearance rate, and long distribution and elimination half-lives make a drug more prone to elimination during TPE. A high frequency and longer duration of the procedure may also contribute to altering a drug’s concentration. The safest choice would be to start and finish TPE before antimicrobial agent infusion. If this not feasible, a reasonable alternative is to avoid administering the drug just before TPE and to delay the procedure for the time of the administered drug’s distributive phase. Ultimately, if plasma exchange must be performed urgently or the drug has a very narrow therapeutic index, monitoring its plasma concentration is advised.

## 1. Introduction

Extracorporeal blood clearance techniques play an important role in treating certain conditions in modern medicine. Therapeutic plasma exchange (TPE) is a procedure in which plasma is separated from the morphotic elements of blood and is then replaced by either albumin solution or fresh frozen plasma (FFP). The aim of TPE is to eliminate morbific factors, often pathological antibodies [1]. However, as plasma removal leads to a decrease in many physiological elements, it is essential to adjust the dose of plasmapheresis to the patient’s capability to resynthesize the lost molecules, i.e., proteins. Furthermore, many individuals undergoing TPE simultaneously require drug administration, in which plasma concentrations can be altered by the procedure, leading to a possible decline in their therapeutic effect [2]. This issue has taken on a key significance as far as the management of infections is concerned. Reliable monitoring of the efficacy of antimicrobial treatment was found to be limited in subjects treated with extracorporeal techniques [3]. As the applied treatment should be effective and safe for the patient, it is necessary to take these possible interactions into account while planning treatment, especially in critically ill subjects.

## 2. Clinical Use

The guidelines of American Society for Apheresis (ASFA) include 87 neurological and non-neurological diseases in which TPE can be implemented [4]. The most common ones that usually require simultaneous treatment in the intensive care unit (ICU) are: severe myasthenia gravis with acute respiratory failure; Guillain–Barré syndrome with acute respiratory failure; Goodpasture syndrome with acute respiratory and/or renal failure; thrombotic thrombocytopenic purpura (TTP) with serious bleeding; acute pancreatitis with extreme hypertriglyceridemia, with acute abdomen syndrome; and severe intoxications with several substances [5]. The indications for TPE are classified into four categories, depending on the quality of evidence of the treatment’s efficacy.

Group I consists of diseases in which TPE is a first-line treatment, namely:

- Myasthenia gravis—removal of anti-AChR and anti-MuSK antibodies;
- Thrombocytopenic purpura—removal of anti-ADAMTS13 IgG autoantibodies;
- Guillian–Barré Syndrome—removal of various autoantibodies against gangliosides including GM1, GD1a, GalNAc-GD1a etc.;
- Wilson’s disease (fulminant)—removal of copper.

Group II considers disorders for which TPE works as an adjunct or a second-line treatment, namely:

- Lambert–Eaton myasthenic syndrome—removal of autoantibodies against the voltage-gated calcium channel (VGCC);
- Systemic lupus erythematosus (severe);
- Myeloma cast nephropathy—removal of light chains (Bence–Jones protein);
- Mushroom poisoning.

In case of Group III, although the role of TPE has not yet been established, theoretical and case report implications for its use exist concerning the following:

- Autoimmune hemolytic anemia—removal of IgG hemolysins;
- Hypertrigliceridemic pancreatitis—lowering triglyceride levels, reduction of inflammatory cytokines, and potential replacement of deficient LpL or apolipoproteins when plasma is used as the replacement fluid;
- Immune thrombocytopenia—removal of autoantibodies against platelet surface antigens, primarily GPIIb/IIIa and/or GPIb/IX;
- Immunoglobulin A nephropathy—removal of pathological IgA and related immune complexes;
- Sepsis with multi-organ failure.

Group IV concerns diseases for which existing data suggest TPE is harmful or ineffective:

- Psoriasis
- Systemic Amyloidosis
- Amyotrophic Lateral Sclerosis
- Polymyositis/dermatomyositis

A full display of the indications and recommendations of ASFA is given elsewhere [4].

Since TPE is a relatively invasive method, several contraindications and side effects exist. Hemodynamic instability and allergy to supplementary fluids (albumin solution, FFP) are the most significant comorbidities that must be taken into account during TPE qualification [6]. Side effects can be associated with both central line placement (infections, bleeding, pneumothorax) and the procedure itself. Most common are anaphylactoid reactions (mostly associated with replacement fluid infusion), citrate toxicity, hypotension and hypocalcemia [7]. Careful clinical assessment and an individualized approach can reduce the occurrence of severe undesirable events.

## 3. Procedure

**Figure 1:**
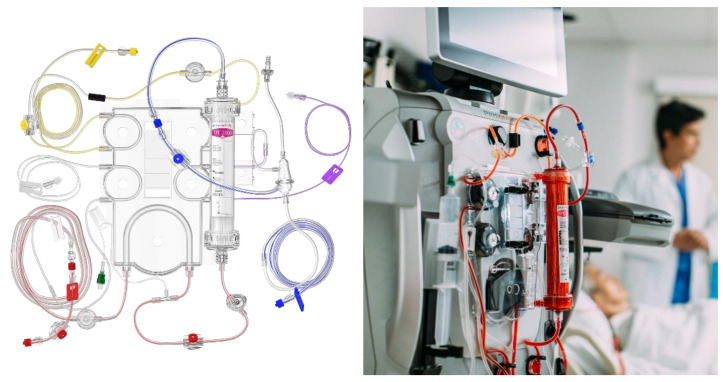
Semipermeable membrane-based plasmapheresis with heparin anticoagulation (Baxter^®^ educational materials): yellow—effluent line, transparent—heparin line, red—arterial line, blue—venous line, purple—replacement fluid line.

**Figure 2:**
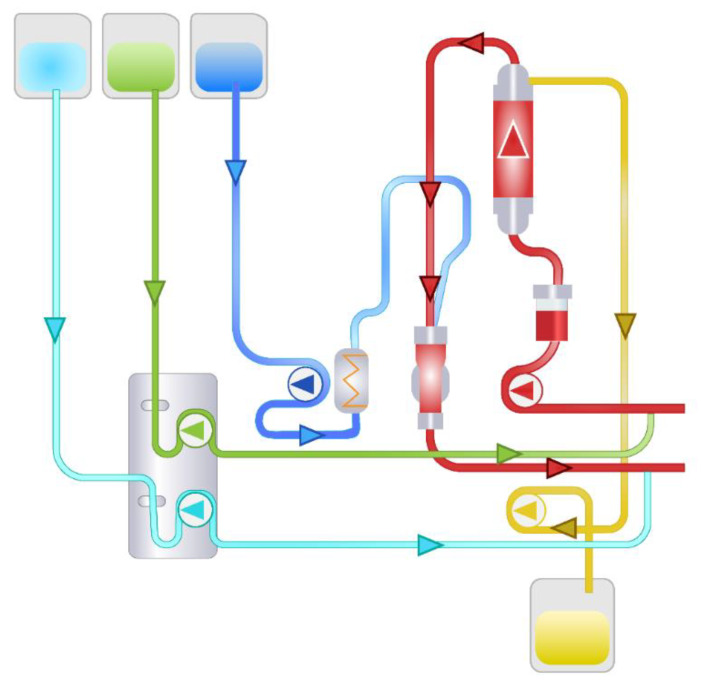
Semipermeable membrane-based plasmapheresis with citrate anticoagulation: light-blue—calcium line, green—citrate line, dark blue—replacement fluid line, yellow—effluent line, red—venous and arterial lines [10].

**Figure 3:**
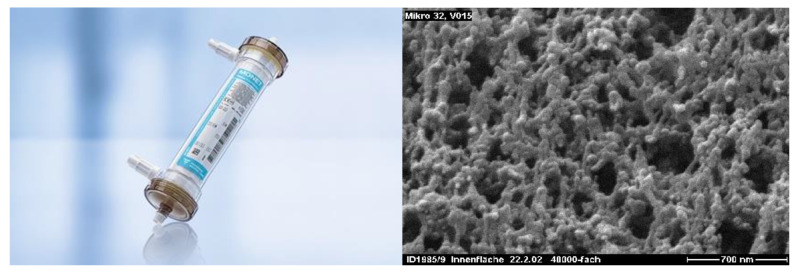
Illustration of MONET^®^ plasmafilter (left, Fresenius Medical Care^®^ educational materials) and a microscopic view of its pores (right, adapted with permission from Rimmelé et al. [13]) used in plasmapheresis: the diameters of pores should be approximately 0.2–0.6 micrones, the total filtration surface of plasmafilter of 0.3–0.6 m^2^ and for molecules with a mass of 15 kDa–2 MD, the permeability ratio should be of high value.

In order to perform TPE, vascular access via a central vein is necessary. The cannula should present certain properties, such as a length of 16–28 cm and a size of 13–14 Fr in order to provide 100–300 mL min^−1^ of blood flow [8]. The patient’s blood is moved from the vascular bed by negative pressure generated by the pumps in the apparatus. Since blood passes through synthetic tubes, anticoagulation is necessary. This can be achieved by systemic (heparin-based) or local (citrate-based) anticoagulation (Figure 1 and Figure 2). The systemic method is based on constant heparin infusion and requires regular monitoring of the activated partial thromboplastin time (APTT), as well as having some possible systemic side effects (related to iatrogenic bleeding diathesis). Heparin-based anticoagulation is still the most frequently used method of anticoagulation. Local anticoagulation comes with a lower risk and fewer side effects regarding hemostasis [9] but is less commonly applied during TPE due to technical reasons [10]. Sodium citrate, infused at the beginning of the tube system, chelates calcium ions (coagulation factor IV), leading to a decrease in their plasma concentration to a level (<0.33/0.4 mmol L^−1^) that inhibits coagulation. Before the blood’s return to the vascular bed, calcium is reinfused to bring back its physiological level. Thanks to this method, anticoagulation is maintained only within the TPE apparatus and the patient’s hemostasis remains undisturbed. It is important to note that citrate may alter the acid-base balance, as well as carrying a caloric load [11]. For the purpose of separating solid elements from the plasma, either a centrifugal cell separator or a semipermeable membrane may be used. The separator draws blood and divides its elements by their relative density, using density gradient centrifugation. If a semipermeable membrane is used, large molecules, i.e., antibodies or albumins (>50–60 kDa), diffuse through its large pores (0.2–0.6 microns), whereas cellular components are not able to pass through (Figure 3) [12,13]. It is vital to note that membrane filtration is the dominant method in Europe [12]. After the blood is separated, it connects with replacement fluid and returns to the patient’s system as a reinfusate. The eliminated plasma is replaced by either albumins or fresh frozen plasma (FFP) [12]. Apart from certain indications, albumin solution is preferred as it presents a lower rate of immunological complications than FFP [14]. In order to compensate for the plasma loss, the exact same amount of replacement fluid must be administered. However, for an individual without any circulatory failure risks, the net fluid balance can be either positive or negative, up to 10–15% of total blood volume (TBV) [12]. TBV is calculated using the following equation: TBV = 70 mL kg^−1^ of body weight. If the hematocrit value (Hct) is taken into account, the total plasma volume can be estimated (plasma = TBV × (1 − Hct)). For a patient body weight of 70 kg and an Hct level of 0.45, plasma volume should be around 2695 mL. The recommended value of eliminated plasma varies depending on the characteristics of the disease. For a single procedure, the guidelines suggest exchanging 1–1.5× of plasma volume. Higher doses come with the risk of excessive reduction of the necessary plasma elements, i.e., coagulation factors [2]. It is therefore necessary to monitor concentrations of the latter—for example, low baseline values of fibrinogen (<140 mg dL^−1^) are an indication for FFP or TPE dose reduction, as otherwise hemostatic balance may be disturbed [12]. Most of the plasma elements are distributed both in the vascular and extravascular compartments meaning that their total body stores are only partially affected by a single dose of TPE. This is caused by the fact that after eliminating certain factors, re-equilibration of the extravascular compartment occurs. Intercompartment equilibration depends on the volume of distribution (Vd): the higher the Vd value, the more significant the redistribution effect. For example, there are differences in distribution between IgM and IgG antibodies. IgM resides mainly in the vascular compartment (75–90% of total body stores) resulting in low Vd values, whereas IgG is located more equally in the compartments within the body (35–45% intravascularly) [15]. Therefore, the redistribution effect in case of IgG removal is more significant—a single dose of plasmapheresis would not be sufficient as, after the procedure, antibodies located outside the vessels would transfer to compensate for the loss. Since one procedure leads to 35% elimination of IgG and 59% of IgM total body stores, it needs to be multiplied. Assuming that 1× of plasma volume is exchanged, the total number of procedures required for total IgG depletion would be six or seven, whereas for IgM it would be around three or four. A similar analysis can be conducted for other molecules that are target of the treatment. However, Vd values are not the only factor accounting for the elimination efficacy, as described below.

## 4. Basics of Pharmacokinetics During TPE

Usually, the drug distribution volume (Vd) and protein-binding affinity have been considered to be the two most significant factors determining drug elimination during TPE. It has been suggested that a low Vd value (<0.2 L kg^−1^) and a high protein-binding affinity rate (>80%) are associated with increased removal [16]. As both of these factors correlate with the drug’s presence in the vascular compartment, they influence the susceptibility to plasma exchange. However, as there are many aspects that modify Vd and protein-binding values, they cannot be assumed as the same in every patient and vary in different populations and conditions [17]. Critically ill patients, especially those with septic shock undergoing aggressive fluid therapy, are vulnerable to drastic Vd changes [18]. Additionally, kidney and liver dysfunction are worth emphasizing: indeed, it has been reported that impaired renal clearance can increase drug level in the vascular compartment [19]. Variations of cardiac output in subjects with sepsis-related cardiac dysfunction is also of great importance. As for factors associated with the drug itself, a different number of dosages can variously alter the Vd value, which also is the case in drug overdose [20]. An endogenous clearance rate of less than 4 mL min^−1^ (commonly observed in acute kidney injury or other severe organ dysfunction), is considered to be a critical value for which TPE alters the drug plasma levels [21]. A normal TPE procedure lasts approximately two hours and, consequently, drugs with a longer elimination half-life (less than two hours) are the most prone to be affected [22]. Moreover, multicompartmental kinetics are increasingly being discussed in this regard. The distribution half-life (T1/2a) describes the time of drug diffusion to different compartments. It has been reported that a longer T1/2a is related with higher elimination rates, mainly due to a drug’s prolonged presence in plasma, including antibiotics [23]. To minimize a drug’s interference with TPE, the clinician should estimate the distributive phase of the administered substance and possibly delay plasmapheresis [24]. The next factor to be considered is the drug level in the removed plasma (plasmapheresate). This is calculated as follows [25]: QPE = CPE × VPE; QPE—amount in plasmapheresate (mg), CPE—drug concentration in plasmapheresate (mg L^−1^), VPE—volume of plasma removed (L).

Total QPE can be used in the calculation of drug clearance due to plasmapheresis, which may also be helpful at adjusting drug doses [25]: CLPE = QPE / AUCPE; CLPE—drug clearance due to TPE, AUCPE—area under the systemic drug concentration versus time curve during plasmapheresis.

CLPE is, however, difficult to establish. It would require collecting multiple blood samples and measuring their drug concentrations in order to assess AUCPE. Drug clearance due to TPE can be used for estimating the infusion rate for drugs during plasmapheresis [26]: IR = (CLE × Css) + CLPE; IR—infusion rate, CLE—endogenous clearance (mg min^−1^), Css—concentration of drug in steady state (mg L^−1^).

There are several limitations to this method. Firstly, there is a limited number of drugs that can be measured in blood and plasmapheresate [24]. Secondly, these considerations do not take the post-redistribution effect into account.

A small number of studies on replacement fluid and its impact on drug elimination exist. Most of the reports discuss cases of drug overdose, for which the use of albumin or FFP can help redistribute the drug to the vascular space and, as a result, make it more available for removal during plasma exchange. Theoretical implications suggest that the lack of albumin supplementation after plasma removal can increase the free fraction of antimicrobial medications after redistribution [22].

## 5. Antimicrobials During TPE

### 5.1. Beta-Lactams

The beta-lactam group contains a wide spectrum of antibacterial drugs: penicillins, cephalosporins, monobactams and carbapenems. Pharmacodynamically, they present similar properties, mainly time-dependent activity [27]. This means that the biological effect of killing bacteria is only maintained only during the time when the concentration of the given substance is above the minimum inhibitory concentration (MIC). Therefore, serial continuous infusion is a preferable method of administration. Endogenous clearance is primarily conducted via the kidneys (with the exception of ceftriaxone and oxacillin) [28,29]. These considerations imply possible interactions in patients undergoing simultaneous beta-lactam therapy and plasma exchange.

### 5.2. Penicilines

#### 5.2.1. Ampicillin

Ampicillin is a semisynthetic aminopenicillin with an average Vd of 0.2–0.3 L kg^−1^ in adults and a protein-binding affinity of around 20%. There is only one study that has explored its relationship with TPE. As this was based on a neonate population, its conclusions are limited. Nonetheless, performing plasmapheresis resulted in a mean decrease of ~35% of total ampicillin concentration [30]. Theoretically, although a low Vd value would account for these findings, the drug presents, on the contrary, a low protein-binding affinity, which suggests a role involving other factors. Even though the evidence coming from the aforementioned study is incomplete, the authors recommended a supplemental dose of the antibiotic once the administration has occurred within six hours of plasma exchange.

#### 5.2.2. Piperacillin

Piperacillin is a part of the ureidopenicillin group with a Vd value of 0.24 L kg^−1^ in all age groups and a protein-binding affinity of around 30%. A single-patient study analyzing the tissue concentrations of the antibiotic (microdialysis) proved that plasma exchange did not alter the serum concentration of the drug administered by continuous infusion, due to enhanced redistribution from the extravascular compartment [31]. Its low protein-binding affinity may account for this finding. Despite the fact, in the presented case, that serum and tissue concentrations were maintained above the MIC level, it is necessary to consider even a slight reduction in tissue concentration in cases of pathogens with high MICs.

No other studies have been performed on penicillins. Nevertheless, theoretical data are available (Table 1).

### 5.3. Cephalosporins

#### 5.3.1. Ceftriaxone

Ceftriaxone is a third-generation cephalosporin with an average Vd value of L kg^−1^ in adults and a T1/2a of 0.23–0.7 h. Although its protein-binding affinity is around 95%, it presents distinctive dose-dependent kinetics (1 g dose has a Vd value of 0.1 L kg^−1^, 96% protein-bound, while 2 and 3 g doses have a Vd value of 0.2 L kg^−1^, 83% protein-bound) [28]. Ceftriaxone endogenous clearance is impaired in patients with severe kidney dysfunction, namely GFR < 10 mL min^−1^. There have been two studies conducted that were focused on the ceftriaxone and TPE relationship [35,36]. The findings were similar—ceftriaxone plasma concentration was significantly modified during TPE. The closer the time between drug administration and TPE initiation, the greater the changes in the plasma ceftriaxone levels. Indeed, the low Vd value and high protein-binding affinity of ceftriaxone indicate its major presence in plasma and, thus, TPE’s role in drug removal. Based on theoretical data and results of the studies, the authors recommended administering ceftriaxone 15 h before or right after TPE.

#### 5.3.2. Ceftazidime

Ceftazidime is a third-generation cephalosporin with an average Vd value of 0.23 L kg^−1^ in adults and a T1/2a of 0.26–0.51 h [37,38]. Its protein-binding affinity is around 10% and it is mainly excreted by the kidneys. A clinical study showed that ceftazidime elimination during TPE was less than 10%, despite renal impairment occurring in several patients. The drug’s low protein-binding affinity is worthy of notice. The recommendation for administration of intramuscular ceftazidime three hours before plasmapheresis vs. the two-hour time interval recommended for intravenous administration is based on the one-hour time interval between intramuscular injections [32]. Based on ceftazidime pharmacokinetic parameters and the above-mentioned study, intravenous administration is recommended two hours before TPE, while the time interval for the intramuscular route must be no less than three hours.

#### 5.3.3. Cefepime

A member of the fourth generation, cefepime presents a Vd value of around 0.32 L kg^−1^ and a distribution half-life of 0.3 h [39]. Its approximate protein-binding affinity is 20% [39]. The drug is mainly excreted via the kidneys and lowers significantly in patients with decreased GFR. TPE’s role in the elimination of cefepime has been analyzed. Researchers measured concentrations of cefepime before and after plasmapheresis—TPE was accounted for removing only ~4% of the total administered dose (2g). The authors suggested that its low binding affinity and quick distribution phase may be responsible for these findings [40].

No other studies have been published on cephalosporines and their relationship with plasmapheresis. Only theoretical data may be advisable (Table 2).

### 5.4. Monobactams

#### Aztreonam

There are no existing data regarding TPE’s influence on monobactam concentrations. Nonetheless, the Vd value of aztreonam is ~0.2 L kg^−1^ and its protein-binding affinity is 56–72%, with a distribution half-life of 0.2 h after intravenous injection [45]. Although it is unknown how simultaneous TPE initiation would alter aztreonam plasma levels, its pharmacokinetic properties suggest that it could be moderately affected.

### 5.5. Carbapenems

#### 5.5.1. Imipenem

No studies have been conducted on imipenem and plasmapheresis. The drug’s Vd value is ~0.23–0.31 L kg^−1^ and it has a protein-binding affinity of approximately 20% [46].

#### 5.5.2. Meropenem

There are no existing data on meropenem and TPE. The drug’s Vd value and protein-binding affinity is 0.25 L kg^−1^ and 2%, respectively [47].

The pharmacokinetics of carbapenems would suggest that plasma exchange would not influence their therapeutic concentrations significantly.

### 5.6. Glycopeptides

#### 5.6.1. Vancomycin

Vancomycin is an antibiotic of glycopeptide group, with a Vd value of 0.4 L kg^−1^, it is ~50% protein-bound. Its distribution half-life is around 0.4–0.94 h [48]. It is mainly excreted via the kidneys (80–90%) [49]. There have been several reports on vancomycin plasma removal [50,51,52]. Although the studies were differently designed, it is likely that vancomycin removal is not clinically significant. Kinztel et al. suggested dose adjustments based on drug concentrations or administration after TPE [32]. Measurement of vancomycin concentrations on a daily basis should be recommended in order to adjust the dose.

#### 5.6.2. Teicoplanin

As one of the glycopeptides, the drug’s steady state Vd value is 0.86 L kg^−1^ and it is approximately 90% protein-bound [53]. Endogenous clearance is maintained via the kidneys. One study explored the pharmacokinetics of teicoplanin while initiating TPE simultaneously [53]—finding that 20% of administered teicoplanin was removed by plasma exchange. The authors recommended a time separation between drug administration and TPE of at least four hours—after this time, the distributive phase of teicoplanin is complete. The drug’s pharmacokinetic properties account for these findings.

### 5.7. Aminoglycosides

Aminoglycosides present mostly a concentration-dependent killing characteristic where their antimicrobial effect is determined by C_max_/MIC [54]. For such agents, the optimal therapeutic action can be expected when the concentrations are ≥10 times above the MIC for the target organism at the site of infection [55]. In order to reach C_max_, it is especially important that no factor interferes with the pharmacokinetics of the administered drug. Another important aspect of aminoglycoside pharmacodynamics is the occurrence of a significant postantibiotic effect (>3 h), which can be described as continuous suppression of bacterial growth after limited exposure to an antibiotic [56]. The long postantibiotic effect allows long dosing intervals, which can be calculated based on the half-life of the aminoglycoside. Such a feature creates an opportunity for establishing a treatment plan in which TPE and antibiotic therapy do not collide.

#### Tobramycin

Tobramycin is an aminoglycoside with a Vd value of 0.33 L kg^−1^ and a protein-binding affinity of less than 10%. Its distribution half-life (T1/2 a) is between 0.1–0.3 h while renal clearance accounts for 90% of its removal [57,58]. These values indicate that it would be only minimally affected by TPE, a fact which has been confirmed in a study performed on two adults in which the researchers measured the actual amount of the removed drug and reported it was around less than 10% of total body stores in both cases [59,60]. Nevertheless, additional reports have presented cases of patients with simultaneous acute kidney injury. In the described patients, the clearance of tobramycin increased more than 30% after plasma exchange while total body stores decreased by approximately 10% [59,60]. Even though the available data and theoretical analysis lead one to consider the influence of TPE on tobramycin levels as insignificant, Kintzel et al. suggested withholding plasmapheresis by at least two distribution half-lives of tobramycin, or performing TPE before drug administration [32].

No other studies have been published on aminoglycosides and their relationship with plasmapheresis (Table 3).

### 5.8. Fluoroquinolones

There are no reports on fluoroquinolones and plasmapheresis. Their pharmacokinetic properties have been listed below (Table 4).

### 5.9. Macrolides

There are no existing data on macrolides and plasmapheresis. Their pharmacokinetic properties have been listed below (Table 5).

### 5.10. Colistin

There are no existing data regarding colistin removal during TPE. Furthermore, pharmacokinetic data are not consistent, as its volume of distribution and protein binding differ drastically in critical states and present large interindividual variability. One study estimated the Vd value of colistin to be around 0.5 L kg^−1^ and 50% protein-bound [68]. The distribution half-life is a T1/2a of 0.5 h. Colistin is predominately eliminated by the kidneys [69]. As it is unknown how TPE would interfere with colistin plasma concentrations, the possible methods of optimizing therapy include monitoring the drug concentration or ending the procedure before colistin infusion.

### 5.11. Antivirals

#### 5.11.1. Acyclovir

Acyclovir is an antiviral guanine analogue, used in treating Herpes simplex and Varicella zoster infections [32]. Its volume of distribution, protein-binding affinity, distribution half-life are approximately 0.69 L kg^−1^, 15% and 0.11–0.26 h, respectively [70,71,72,73]. It is predominantly excreted by the kidneys [73]. There have been studies in which the administration of acyclovir occurred one to three hours before TPE. Moreover, 2.5% of total acyclovir systemic stores were eliminated via plasmapheresis [74]. Despite the clinical insignificance of this finding, it has been suggested postponing TPE for at least three hours after acyclovir infusion [32].

#### 5.11.2. Oseltamivir

There are no reports concerning the relationship of oseltamivir with plasmapheresis. The drug’s Vd value is 0.32–0.37 L kg^−1^ while its protein-binding affinity is approximately 42%. Its metabolite is mainly (90%) excreted via the kidneys [75]. Although it is unknown how TPE would interfere with oseltamivir plasma concentrations, pharmacokinetic considerations rather imply the insignificance of plasma exchange.

### 5.12. Antifungals

#### 5.12.1. Amphotericin B (liposomal)

Amphotericin B is a monocyclic, polyene antifungal drug with a Vd value of 0.1–0.2 L kg^−1^ while 95–99% of the drug is protein-bound, mainly to LDL, albumin and α-1-acid glycoprotein [76]. Treatment efficacy depends on achieving a concentration above MIC [76]. There is only one report in which a patient with liposomal amphotericin B treatment received plasmapheresis. Pre-TPE plasma concentration of the drug was 0.5 μg mL^−1^ and post-TPE concentration was 0.3 μg mL^−1^. This means that the reduction ratio was 40% and resulted it in falling below MIC. The authors recommended frequent amphotericin B plasma level monitoring and adjusting the doses to assure the effectiveness of the antifungal therapy [77].

#### 5.12.2. Voriconazole

Voriconazole is a broad-spectrum antifungal agent with a Vd value of 4.5 L kg^−1^ and a protein-binding affinity of 58% [78]. There has been a report of a critically ill patient with fungal pneumonia that required plasmapheresis due to an underlying condition. The authors administered voriconazole 2.5 h before TPE initiation. The effect of plasmapheresis on the drug levels was clinically insignificant, which is compatible with the theoretical pharmacokinetic properties of voriconazole [78].

No other studies have been published on antifungals and their relationship with plasmapheresis (Table 6).

### 5.13. Other Antimicrobials

The prediction of TPE influence on other antimicrobial agents is depicted in Table 7.

## 6. Conclusions

**Figure 4:**
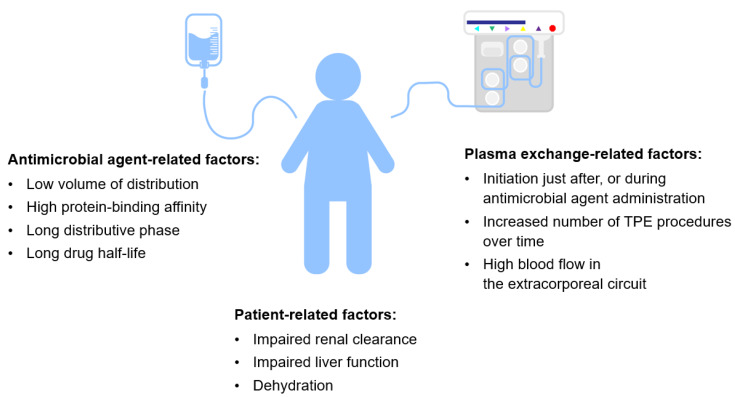
Possible factors accounting for reduction of antimicrobial treatment efficacy.

Antimicrobial treatment during TPE remains a complex issue (Figure 4). Recommendations based on a limited number of experimental studies should be implemented in clinical practice with caution. Effective management of infections due to plasma or albumin-related interactions, as well as impaired pharmacokinetics, in critical illness is difficult. Nevertheless, knowing the pharmacokinetics of the drugs concerned and the procedural aspects of plasmapheresis should be helpful in planning personalized treatment. In general, possessing a low distribution volume, a high protein-binding affinity, a low endogenous clearance rate, and long distribution and elimination half-lives make a drug more prone to elimination during TPE. A high frequency and longer duration of the procedure may also contribute to altering a drug’s concentration. The safest choice would be to start and finish TPE before antimicrobial agent infusion. If this not feasible, a reasonable alternative is to avoid administering the drug just before TPE and to delay the procedure for the time of the administered drug’s distributive phase. Ultimately, if plasma exchange must be performed urgently or the drug has a very narrow therapeutic index, monitoring its plasma concentration is advised. In conclusion, antimicrobial treatment would be effective during therapeutic plasma exchange only if planned correctly. Moreover, the paucity of up-to-date data should encourage researchers to explore this complex issue using case-series and observational surveys.

## Figures and Tables

**Table 1:** Therapeutic plasma exchange (TPE) and penicillins.

Antibiotic	Distribution Volume [L kg^−1^]	Protein Binding [%]	Distribution Half-Life [min]	Renal Clearance [%]	Prediction of TPE Influence
Ampicillin [30]	0.2–0.3	20%	N/A	60–80	moderate
Amoxicillin [32]	0.21	18	N/A	68	moderate/insignificant
Penicillin G [33]	0.53–0.67	45-68	N/A	60–90	insignificant
Ticarcillin [34]	0.17–0.23	45–65	N/A	60–70	moderate/insignificant
Piperacillin [28]	0.24	30%	N/A	68	insignificant

N/A—not available.

**Table 2:** TPE and cephalosporins.

Antibiotic	Distribution Volume [L kg^−1^]	Protein Binding [%]	Distribution Half-Life [min]	Renal Clearance [%]	Prediction of TPE Influence
Cefazolin (1st gen.) [41]	0.19	88	N/A	80	significant
Cefuroxime (2nd gen.) [42]	0.2	33–50	N/A	96	moderate/insignificant
Ceftazidime [37]	0.23	10	16–31	99	moderate/insignificant
Ceftriaxone [28]	0.13	95	14–42	50–60	significant
Cefotaxime (3rd gen) [43]	0.23	30	N/A	50	insignificant
Cefepime [39]	0.32	20	18	85	insignificant
Ceftaroline (5th gen) [44]	0.37	20	N/A	88	insignificant

N/A—not available

**Table 3:** TPE and aminoglycosides.

Antibiotic	Distribution Volume [L kg^−1^]	Protein Binding [%]	Distribution Half-Life [min]	Renal Clearance [%]	Prediction Of TPE Influence
Amikacin [61]	0.27	4	N/A	>90	insignificant
Gentamicin [62]	0.25	0–30	21–41	>90	insignificant
Streptomycin [63]	0.26	35	N/A	>90	insignificant
Tobramycin [57,58]	0.33	<10	6–20	90	insignificant
Kanamycin [32]	0.19	0–3	N/A	>90	moderate/insignificant
Netilmycin [32]	0.16–0.34	0–30	N/A	>80	moderate/insignificant

N/A—not available.

**Table 4:** TPE and fluoroquinolones.

Antibiotic	Distribution Volume [L kg^−1^]	Protein Binding [%]	Distribution Half-Life [min]	Renal Clearance [%]	Prediction of TPE Influence
Ciprofloxacin [64]	2–3	20–30	N/A	~70	insignificant
Levofloxacin [65]	1.1	30–40	27	>85	insignificant
Moxifloxacin [32]	2	30–40	N/A	~40	insignificant
Ofloxacin [32]	1.8	25	N/A	~90	insignificant
Norfloxacin [32]	0.36–0.5	10–15	N/A	~65	insignificant

N/A—not available.

**Table 5:** TPE and macrolides.

Antibiotic	Distribution Volume [L kg^−1^]	Protein Binding [%]	Distribution Half-Life [min]	Renal Clearance [%]	Prediction of TPE Influence
Azithromycin [66]	0.44	12–52	N/A	12–20	insignificant
Clarithromycin [67]	2.6	70	N/A	15–30	insignificant
Erythromycin [32]	0.78	84	N/A	2.5	moderate/insignificant

N/A—not available.

**Table 6:** TPE and antifungal agents.

Antibiotic	Distribution Volume [L kg^−1^]	Protein Binding [%]	Distribution Half-Life [min]	Renal Clearance [%]	Prediction of TPE Influence
Amphotericin B [73]	0.1–0.2	95–99	N/A	N/A	significant
Ketoconazole [32]	2.4	99	N/A	13	moderate/significant
Fluconazole [32]	0.6	11	N/A	80	insignificant
Voriconazole [32]	4.5	58	N/A	94	insignificant
Terbinafine [32]	>29	99	N/A	N/A	moderate/insignificant
Caspofungin [32]	0.3–2	97	N/A	41	moderate/significant

N/A—not available.

**Table 7:** TPE and other popular antimicrobial agents.

Antibiotic	Distribution Volume [L kg^−1^]	Protein Binding [%]	Distribution Half-Life [min]	Renal Clearance [%]	Prediction of TPE Influence
Metronidazole [79]	0.25–0.85	<20	N/A	60–80	insignificant
Clindamycin [80]	1.1	60–94	N/A	~33	moderate/insignificant
Sulfamethoxazole [81]	0.43	70	N/A	84.5	moderate/insignificant
Trimethoprim [81]	0.7–1.5	50	N/A	66.8	insignificant
Linezolid [82]	0.57–0.86	31	N/A	35	insignificant

N/A—not available.
